# Supplementary material for: k-mer manifold approximation and projection for visualizing DNA sequences
Source: Genome Res. 2025 May;35(5):1234–46. doi: 10.1101/gr.279458.124 (PMC12047656; doi:10.1101/gr.279458.124)
Supplement: Supplement 8 [file Supplemental_Data_1.zip › Supplemental_Data_1/HOXC11_TGCGAA20NGA_AF_4/HOXC11_TGCGAA20NGA_AF_4_MDS.pdf]

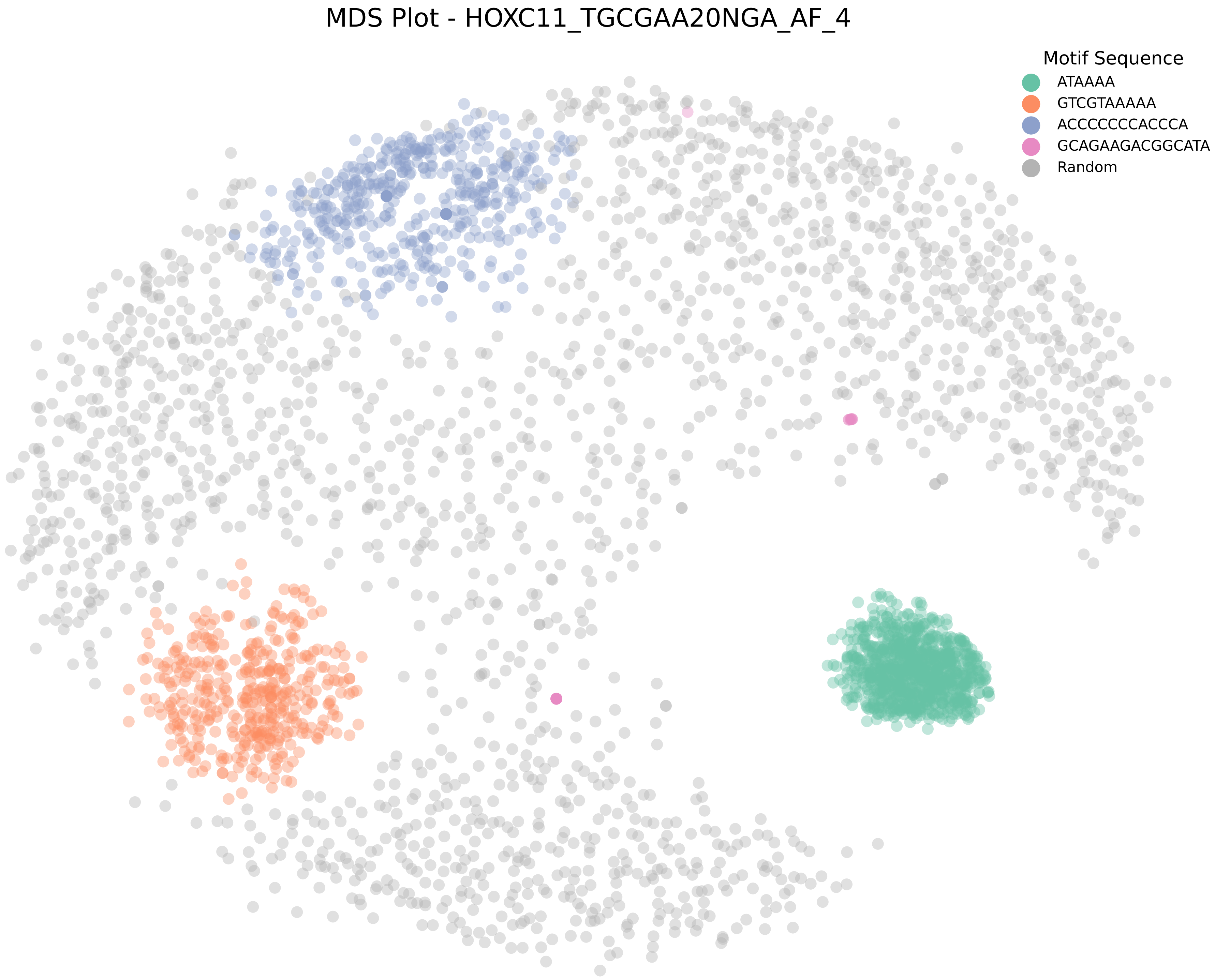
MDS Plot - HOXC11_TGCGAA20NGA_AF_4
Motif Sequence
ATAAAA
GTCGTAAAAA
ACCCCCCCCACCCA
GCAGAAGACGGCATA
Random
